# Implants in grafted and native bone in patients with ectodermal dysplasia

**DOI:** 10.1186/1746-160X-8-S1-O2

**Published:** 2012-05-25

**Authors:** M Callea, F Grecchi, F Carinci, EG Mancini

**Affiliations:** 1 Department of Maxillo-Facial Surgery and Paediatric Dentistry, Institute for Maternal and Child Health, Trieste, Italy; 2 Galeazzi Hospital, Milan, Italy; 3 University of Ferrara, Italy

## 

Ectodermal dysplasia (ED) is a congenital syndrome characterized by abnormalities of tissues of ectodermal origin, namely skin, nails, hair, and teeth. Dental treatment of patients with ED is necessary because it provides the opportunity to develop normal speech, chewing, swallowing, and facial support. Because there are few reports on implants inserted in grafted bone in patients with ED, we performed a retrospective study on 44 implants in 4 patients to determine variables that affect survival and crestal bone remodeling around the implant neck in such subjects. Forty-four fixtures were analyzed. Several patient-related (age and sex), anatomic (maxilla, mandible, tooth site), implant (type, length, diameter), surgical (sites and types of grafts), and prosthetic variables (type of loading) were investigated. Implant failure and peri-implant bone resorption were considered as predictors of clinical outcome. Kaplan-Meier algorithm and Cox regression analysis were then performed to detect those variables that are associated with clinical outcome. Implant length and diameter ranged from 11.5 to 15 mm and from 3.5 to 4.0 mm, respectively. Implants were inserted to replace 12 incisors, 12 cuspids, 11 premolars, and 9 molars. No implant was lost. Particular importance of implant length, graft sites, and type of loading was shown by univariate analysis, but these data were not confirmed by multivariate algorithm. In ED patients, dental implants and bone grafts proved to be valuable treatment options which are as effective as in patients not affected by ED, at least in adults. We also analysed the choice to conserve all teeth present in the frontal region. Orthognatic rehabilitation prior to any intervention has been the paramount criterion. In molar regions with tooth agenesis, placement of implants required bypassing of the Nervus mandibularis. In the interforaminal sector, bone reconstruction was achieved by bone grafting surgery.

